# Teilhabeberichterstattung in Deutschland: Entwicklung, Wissensbasis und Grenzen nach der Ratifizierung der UN-Behindertenrechtskonvention

**DOI:** 10.1007/s00103-026-04276-w

**Published:** 2026-07-13

**Authors:** Elisabeth Wacker

**Affiliations:** School of Medicine and Health (MH), Department Health and Sport Sciences, Diversitätssoziologie, Technische Universität München (TUM), Am Olympiacampus 11, 80809 München, Deutschland

**Keywords:** Behinderungsvielfalt, Inklusionsziele, Teilhabeberichterstattung, Teilhabesurvey, Ungleichheit, Diversity of disabilities, Inclusion targets, Participation reporting, Participation survey, Inequality

## Abstract

Mit der Ratifizierung der Behindertenrechtskonvention der Vereinten Nationen (UN-BRK) im Jahr 2009 hat sich Deutschland verpflichtet, die Teilhabe von Menschen mit Behinderung systematisch zu fördern und darüber regelmäßig Bericht zu erstatten. Laut Nationalem Aktionsplan soll die Datenlage zu Menschen mit Beeinträchtigungen und Behinderung verbessert werden, wobei die menschenrechtliche Perspektive der Konvention besonders zu beachten ist. Bisher wurden drei entsprechend konzipierte Berichte mit dem programmatischen Titel „Teilhabebericht der Bundesregierung über die Lebenslagen von Menschen mit Beeinträchtigungen. Teilhabe – Beeinträchtigung – Behinderung“ vorgelegt, ein vierter liegt als Entwurf vor.

Diese Berichterstattung flankiert die nach Ratifizierung der UN-BRK deutschlandweit obligatorische Transformation zur inklusionsorientierten Eingliederungshilfe und Rehabilitation. Als werte- und wirkungsorientierte Berichterstattung kann sie dazu mit differenzierter und diversitätssensibler Datenqualität wesentlich beitragen. Entsprechend solides Wissen soll nun mit dem Teilhabesurvey weiter ausgebaut und laufend verbessert werden.

Die vorliegende narrative Übersicht zeichnet die Entwicklung der Teilhabeberichterstattung von der normativen und institutionellen Grundlegung bis zur aktuellen Daten- und Transformationsphase nach. Sie ordnet die Funktionen, Grenzen und offenen Baustellen der Berichterstattung und fragt, ob Berichte tatsächlich Teilhabe verbessern. Dazu werden begriffliche Grundlagen geklärt, methodische Entwicklungen und Datenprobleme analysiert, Spannungen zwischen unterschiedlichen Behinderungsverständnissen beleuchtet, aber auch die institutionellen Rollen dargestellt.

## Abstract (en)

By ratifying the United Nations Convention on the Rights of Persons with Disabilities (UN CRPD) in 2009, Germany undertook to systematically promote the participation of people with disabilities and to report on this regularly. According to the National Action Plan, the data on people with impairments and disabilities are to be improved, with particular attention being paid to the human rights perspective set out in the Convention. To date, three reports designed in accordance with this approach have been published under the programmatic title “Federal Government Report on the Living Conditions of People with Disability: Participation—Impairment—Disability”; a fourth is currently available as a draft.

This reporting supports the transition to inclusion-oriented social integration assistance (Eingliederungshilfe) and rehabilitation, which has become mandatory throughout Germany following the ratification of the UN Convention on the Rights of Persons with Disabilities. As value- and impact-oriented reporting, it can make a significant contribution to this through nuanced and diversity-sensitive data quality. The participation survey is now intended to further expand and continuously improve this solid body of knowledge.

This narrative overview traces the development of Participation Reporting, from its normative and institutional foundations through to the current phase of data collection and transformation. It examines the functions, limitations, and unresolved issues of such reporting and asks whether reports actually improve participation. To this end, it clarifies the conceptual foundations, analyses methodological developments and data issues, highlights tensions between different understandings of disability, and outlines the institutional roles involved.

## Teilhabeberichte – Wirkung und Wandel

Kann die neue Berichterstattung zu Behinderung Teilhabechancen verbessern? Diese aktuelle Frage wird häufig gestellt im Kontext von Klagen über Datenlücken oder der Suche nach Teilhabebrücken [1]. Im Lauf der vergangenen Dekade entwickelten sich zur Teilhabeförderung Visionen und Pragmatiken, mit neuen strukturellen und materiellen Zugängen zu Beeinträchtigungen und Behinderung.

Dabei erscheinen Inklusionsziele relevant, wenn auch bisweilen wie eine Fata Morgana – gleichermaßen unwirklich und zutreffend. Dies geschieht nicht irrtümlich, sondern als eine Folge des *sozialen Modells* von Behinderung, welches sich je nach Perspektive soziokulturell interpretieren, menschenrechtlich einfordern oder ökonomisch nach Machbarkeit beurteilen lässt. Entsprechend entstehen hochkomplexe Fragen zu Beeinträchtigungen und Behinderung, die in einer multiperspektivischen Betrachtung aber wie ein Navigationssystem Wege weisen können. Denn differenzierte Analysen bringen hilfreiches Wissen hervor, wenn sie Vernunft (das Reale), Fantasie (das Erwünschte) und gerechte Teilhabechancen (das Mögliche) vereinen.

Bericht erstatten zur Teilhabe meint die systematische Erfassung und Darstellung der Lebenssituation von Menschen mit Beeinträchtigungen und Behinderung. Sie benennt zunächst konkret, wie für diese Personengruppen beispielsweise Teilhabe am gesellschaftlichen Leben möglich ist. Zugleich kann sich mehr Aufmerksamkeit auf Barrieren richten, die Teilhabe erschweren und (politisches) Handeln erfordern.

Teilhabeverpflichtungen reichen von der Kinder- und Jugendhilfe über den Arbeitsmarkt und die Wohnungslosenhilfe bis in die Pflegegestaltung [1]. Oft erscheinen im politischen und sozialen Alltagsgeschehen Teilhaberechte eher verschwommen, etwa bezogen auf Schulwege oder Arztbesuche von Menschen, die mit Behinderung leben. Zudem oszillieren sie föderal.

In Deutschland wurde die Teilhabeberichterstattung mit der Ratifizierung der Behindertenrechtskonvention der Vereinten Nationen (UN-BRK) im Jahr 2009 grundlegend reformiert. Der Nationale Aktionsplan (NAP) der Bundesregierung legte fest, die Datenlage zu Menschen mit Beeinträchtigungen zu verbessern und dabei die menschenrechtliche Perspektive der Konvention zu beachten. Seitdem erscheinen Teilhabeberichte unter dem programmatischen Titel „Teilhabebericht der Bundesregierung über die Lebenslagen von Menschen mit Beeinträchtigungen. Teilhabe – Beeinträchtigung – Behinderung“.

Diese Leitplanken werden flankiert von Verbesserungswünschen. Gute Gründe dafür sind mehrere Defizite:

- *Datenlücken*: Vorhandene Statistiken und Erhebungen erfassen Menschen mit Behinderung nur unzureichend und in Bezug auf besondere Wohnformen kaum.
- *Definitionsprobleme*: Unterschiedliche Behinderungsdefinitionen führen zu erheblich abweichenden Angaben über die Anzahl und Lage von Menschen mit Behinderung.
- *Menschenrechtlicher Anspruch*: Die UN-BRK fordert einen Perspektivenwechsel vom medizinischen zum sozialen und menschenrechtlichen Verständnis von Behinderung.
- *Wirkungsorientierung*: Berichterstattung soll nicht nur beschreiben, sondern auch zeigen, ob die intendierte wachsende Inklusivität besser gelingt.

Die Vielfalt von Politikfeldern, Rechtsauslegungen, Wissensformen und Lebenslagen fordert heraus, sie differenziert nach wissenschaftlichem, werteorientiertem oder sozialpolitischem Wissen zu ordnen, scheint aber der Mühe wert. Zudem sitzt allerdings „Behinderungswissen“ im Alltagsbewusstsein, ebenso wie beim konkreten Handeln in ökonomischen, politischen und soziokulturellen Ungleichheitsfeldern. Externe Faktoren erweitern also die bestehenden Fragen zu Vielfaltsdimensionen, Lebensbedingungen, Wohlergehen, Selbstbestimmung, Anerkennung und Exklusion.

In diesem narrativen Übersichtartikel wird beschrieben, wie sich die Teilhabeberichterstattung in Deutschland seit der Ratifizierung der UN-BRK entwickelt hat, welche Wissensgrundlagen sie nutzt und wo ihre methodischen und normativen Grenzen liegen und inwiefern sie ein Mittel zur Inklusionsorientierung sein kann. Zunächst werden Begriffe und Ziele der Teilhabeberichterstattung erklärt und historisch eingebettet. Dann werden Rollen beschrieben und Akteure der Berichterstattung gewürdigt, gefolgt von Aspekten zur methodischen Entwicklung, zu Datenproblemen und inhaltlichen Spannungen. Fazit ist, Steuerung über Teilhabeberichterstattung steht auf dem Prüfstand. Dies gilt analytisch und strategisch betrachtet mit Blick auf eine Welt der Rehabilitation und Eingliederungshilfe im Wandel.

## Begriffe, Klassifikation und Ziele

### Teilhabe, Partizipation und Inklusion

Die Begriffe Teilhabe, Partizipation und Inklusion werden häufig synonym verwendet, bezeichnen aber unterschiedliche Aspekte:

- *Teilhabe* meint die Möglichkeit, am gesellschaftlichen Leben in allen Bereichen (wie Bildung, Arbeit, Wohnen, Gesundheit, Freizeit, Politik) gleichberechtigt mitzuwirken.
- *Partizipation* betont die aktive Beteiligung an Entscheidungsprozessen, die das eigene Leben betreffen.
- *Inklusion* geht aus vom gesamten Format einer Gesellschaft, in der alle Menschen selbstverständlich dazugehören und Verschiedenheit als Normalität gilt. Zugehörigkeit ist also bedingungslos vorgegeben ohne vorausgehende Anpassungspflicht. Insofern ist Inklusion der umfassendste Begriff: Sie soll gelingen über den Zugang zu bestehenden Strukturen, vor allem aber auch über deren Veränderung, über den Abbau von Barrieren und die Anerkennung von Verschiedenheit ohne Benachteiligung.

Die UN-BRK bietet Prüfsteine für Aussagen zu gerechten Teilhabechancen [2].

### Beeinträchtigung und Behinderung

Die Teilhabeberichte unterscheiden konsequent zwischen Beeinträchtigungen und Behinderung:

- *Beeinträchtigung* bezeichnet konkrete Einschränkungen bei Aktivitäten in verschiedenen Lebensbereichen – etwa körperliche, sensorische, kognitive oder psychische Funktionseinschränkungen [3, S. 7].
- *Behinderung* entsteht, wenn Beeinträchtigungen mit Barrieren im Umfeld (physisch, kommunikativ, institutionell, attitudinal) so zusammenwirken, dass eine gleichberechtigte Teilhabe eingeschränkt wird [4, S. 22].

Diese Unterscheidung folgt dem *sozialen Modell* von Behinderung, das die UN-BRK prägt: Behinderung ist kein individuelles Defizit, sondern entsteht im Zusammenspiel von Person und Umwelt. Die Verantwortung für Inklusion liegt damit nicht bei den Betroffenen, sondern bei der Gesellschaft und all ihren Mitgliedern insgesamt.

Zusätzlich wird unterschieden zwischen:

- Menschen mit Beeinträchtigungen (unabhängig von einer amtlichen Feststellung),
- Menschen mit (amtlich) anerkannter Behinderung,
- Menschen mit anerkannter Schwerbehinderung (Grad der Behinderung ab 50; [5, S. 25 ff.]).

Diese Differenzierung ist wichtig, weil amtliche Statistiken oft nur Menschen mit anerkannter Behinderung erfassen und die tatsächliche Teilhabesituation breiter Bevölkerungsgruppen verborgen bleibt.

### Der normative Rahmen: UN-Behindertenrechtskonvention

Die UN-Behindertenrechtskonvention (UN-BRK), englisch Convention on the Rights of Persons with Disabilities (CRPD), ist das zentrale menschenrechtliche Referenzdokument für die Teilhabeberichterstattung.

Die Konvention

- bekräftigt, dass Menschen mit Behinderung die gleichen Rechte wie Menschen ohne Behinderung beanspruchen können,
- fordert einen Perspektivenwechsel vom medizinischen zum sozialen und menschenrechtlichen Verständnis von Behinderung,
- verpflichtet die Vertragsstaaten zu konkreten Maßnahmen in allen Lebensbereichen (Artikel 5–30),
- erwartet die Erhebung geeigneter Daten und Statistiken (Artikel 31),
- verlangt regelmäßige Staatenberichte und unabhängiges Monitoring.

Mithilfe der UN-Übereinkunft lassen sich neue Wissensbestände erschließen, die die Teilhabelage für die Bundesrepublik Deutschland ordnen, prüfen, erklären oder ergänzen.

Prinzipiell findet sich in dieser Rahmung für die Lebensspanne jedes Menschen ein Navigationsprofil, das datenbasiert relevante Wechselbeziehungen beachtet und dabei klärt, was zu Behinderung führen kann. Dieses Vorgehen stützt Diskurse und damit eine neue Analyse und Verständigung zu gewohnten Datenbeständen.

### Der Nationale Aktionsplan

Der Nationale Aktionsplan (NAP) „Unser Weg in eine inklusive Gesellschaft“ wurde 2011 vom Bundesministerium für Arbeit und Soziales (BMAS) verabschiedet [6]. Er bündelt Maßnahmen der Bundesregierung zur Umsetzung der UN-BRK und bildet den strategischen Rahmen für die Teilhabeberichterstattung.

Der NAP definiert Handlungsfelder, Ziele und Indikatoren und wird fortgeschrieben. Er sieht vor:

- die Entwicklung inklusionsorientierter Berichtsformate,
- die wissenschaftliche Evaluation von Maßnahmen,
- die Einbindung von Stakeholdern (Verbände, Selbstvertretungsorganisationen, Wissenschaft) [7].

Schwerpunktplanungen sollen datenbasiert weitere Bezüge zur UN-BRK aufbereiten, Meilensteine (Ober- und Zwischenziele) konstruieren und den NAP weiterentwickeln [8, S. 5 f.]. Dabei soll behinderungsspezifisch mehr Inklusivität erreichbar, beobachtbar, berichtbar und bewertbar werden, gestützt von einem wissenschaftlich evaluierten Referenzrahmen zur Wirksamkeit [8].

### Die Internationale Klassifikation der Funktionsfähigkeit, Behinderung und Gesundheit (ICF)

Mit der ICF der Weltgesundheitsorganisation (WHO) steht ein konzeptioneller Rahmen zur Verfügung, um Behinderung biopsychosozial zu erfassen [9]. Sie unterscheidet:

- *Körperfunktionen und -strukturen* (biologische Ebene),
- *Aktivitäten* (individuelle Handlungsfähigkeit),
- *Partizipation* (Teilhabe am gesellschaftlichen Leben),
- *Kontextfaktoren* (personenbezogen und umweltbezogen).

Die ICF ermöglicht internationale Vergleichbarkeit und lenkt den Blick auf das Zusammenwirken von Person und Umwelt. In Deutschland wurde sie auch in sozialrechtliche Regelungen übernommen (SGB IX; [10]). Inklusionsziele bezogen auf Behinderung erfordern es, gerechte Chancen auf Lebensqualität zu sichern und Verschiedenheit anzuerkennen. Für Differenzierungen müssen alle gesellschaftlichen Teilbereiche betrachtet werden – wozu sich die biopsychosoziale Ausrichtung der ICF anbietet, die auch Kontexte einbezieht [5, S. 20–27].

Die biopsychosozial ausgerichtete Klassifikation wird zusätzlich angereichert um wertebasierte Selbstbestimmungs- und Freiheitsrechte. So sollen Diversitätskompetenz und Chancen wachsen, um Gesundheits- und Teilhaberisiken zu reduzieren oder präventiv zu vermeiden.

## Historie der Teilhabeberichterstattung

### Von der Berichtspflicht zur inklusionsorientierten Berichterstattung

Wichtige Meilensteine der historischen Entwicklung der Teilhabeberichterstattung sind in Tab. 1 dargestellt.

**Tab. 1:** Wichtige Meilensteine der Teilhabeberichterstattung

Jahr	Ereignis
1981	UN-Jahr der Behinderten; Deutschland verpflichtet sich zu regelmäßiger Berichterstattung
1994	Grundgesetzänderung: „Niemand darf wegen seiner Behinderung benachteiligt werden“ (Art. 3 Abs. 3 GG)
2006	Verabschiedung der UN-Behindertenrechtskonvention
2009	Ratifizierung der UN-BRK durch Deutschland
2011	Erster Nationaler Aktionsplan (NAP); Initialbericht an den UN-Fachausschuss; Beginn der Vorstudien zum Teilhabesurvey
2013	Erster Teilhabebericht der Bundesregierung
2015	Abschließende Bemerkungen des UN-Fachausschusses zum Initialbericht
2016	Zweiter Teilhabebericht; Bundesteilhabegesetz (BTHG) reformiert SGB IX
2017–2020	1. Welle des Teilhabesurveys
2019	Zweiter und Dritter Staatenbericht an den UN-Fachausschuss
2021	Dritter Teilhabebericht
2023	Abschließende Bemerkungen des UN-Fachausschusses; Maßnahmenbericht
2023/24	2. Welle des Teilhabesurveys
2025	Vierter Teilhabebericht (Entwurf)
2031	Vierter bis sechster Staatenbericht fällig

Die Berichtspflicht zu Behinderung und Teilhabe besteht in Deutschland seit den 1980er-Jahren. Kurz nachdem die UN 1981 das „Internationale Jahr der Behinderten“ ausgerufen hatten, hat Deutschland beschlossen, in jeder Wahlperiode über die Lage der behinderten Menschen und die Entwicklung ihrer Teilhabe zu berichten. So begann eine immer differenziertere Suche nach Teilhabechancen. Die frühen Berichte konzentrierten sich auf Statistiken zu Schwerbehinderten und Leistungen der Rehabilitation [11, 12], ohne systematisch Teilhabe in allen Lebensbereichen zu beachten. Mit der Ratifizierung der UN-BRK änderte sich der Anspruch grundlegend. Nun soll die Berichterstattung

- die *menschenrechtliche Perspektive* der Konvention widerspiegeln,
- den *Perspektivenwechsel* vom Behindert-Sein zum Behindert-Werden abbilden, wobei vor allem gesellschaftliche Barrieren im Fokus stehen [3, S. 7],
- *wirkungsorientiert* sein, also statt nur Maßnahmen aufzulisten, deren Effekte auf Teilhabe zeigen,
- *Datenlücken* schließen und eine differenzierte, diversitätssensible Datenqualität erreichen.

Die Verantwortung für die nationale Umsetzung (National Focal Point) erhielt das Bundesministerium für Arbeit und Soziales (BMAS). Die Monitoring-Stelle am Deutschen Institut für Menschenrechte überwachte unabhängig Teilhabepläne und -ziele, ein Inklusionsbeirat bot aus der Zivilgesellschaft Rat an.

### Berichten als Inklusionsgeleit

Die neue Berichterstattung wollte unter anderem inklusionsorientiert und wirkungsbezogen Fortschritte sichtbar machen [1]. Dies erfordert jedoch den Sozialraum zu betreten, um jenseits etablierter Strukturen der Eingliederungshilfe Wissen zu Beeinträchtigungen und Behinderung neu zu kalibrieren, zu kommunizieren, solide zu verankern und zu bewerten. Dabei erschließt sich gemeindebasiert die vielfache Relativität und Relationalität von Behinderung [13].

Mit dem Motto „einfach machen“ startete das BMAS nationale Transformationsaufgaben, rief zu „einfach teilhaben“ auf und bot Informationen, Inklusionstage, Preisverleihungen und Ratgeber an.^1^

Inklusionsorientierte Berichtsformate trafen nun auf naturhaft gegebene Unterscheidungen von Beeinträchtigungen (biologisch oder nach Funktionalität) und auf soziokulturell geprägte Kategorisierungen (Humandifferenzierung nach Diversitätsdimensionen). Soziale Aspekte wie Zugehörigkeit (mit Selbstzuordnung) und Zusammenhalt (in sozialen Gebilden) warfen weitere Fragen auf, wie sich Teilhabe bei Differenz beschreiben lässt [14].

### Die bisherigen Teilhabeberichte

Alle Teilhabeberichte wurden vom BMAS herausgegeben und folgen einer gemeinsamen lebenslagenorientierten Grundstruktur [3–5]. Zudem setzen sie jeweils thematische Schwerpunkte (Tab. 2) und betrachten Teilhabe in zentralen Lebensbereichen:

- Familie und soziales Netz,
- Bildung und Ausbildung,
- Erwerbstätigkeit und materielle Lebenssituation,
- alltägliche Lebensführung (Wohnen, Barrierefreiheit im öffentlichen Raum, Leistungen für Teilhabe am alltäglichen Leben),
- Gesundheit,
- Freizeit, Kultur und Sport,
- Sicherheit und Schutz der Person,
- politische und gesellschaftliche Teilhabe.

**Tab. 2:** Teilhabeberichte, die vom Bundesministerium für Arbeit und Soziales (*BMAS*) herausgegeben wurden, mit thematischen Schwerpunkten

Bericht	Jahr	Seiten	Schwerpunkte
Teilhabebericht [3]	2013	471	Einführung des neuen Berichtskonzepts; Etablierung der Unterscheidung Beeinträchtigungen/Behinderung
Zweiter Teilhabebericht [4]	2016	573	Vertiefung der Datengrundlagen; Vorbereitung auf den Teilhabesurvey
Dritter Teilhabebericht [5]	2021	828	Schwerpunkt Gesundheit (Kapitel 7 und 8); Erfahrungen aus der Corona-Pandemie; erste Daten aus dem Teilhabesurvey
Vierter Teilhabebericht	(Entwurf)	–	(Teilweise) Integration von Daten aus der 2. Welle des Teilhabesurveys

Bei konstanter Grundstruktur wurden manche Bezeichnungen ergänzt oder modifiziert. Zusätzlich setzte jeder Teilhabebericht zwei ausführlicher dargestellte Schwerpunkte (Tab. 2). Im Schwerpunktbereich „Gesundheit“ des dritten Berichts wurden zwei Zugänge beleuchtet: mit Bezug zu den Artikeln 25 und 26 UN-BRK (Kapitel 7) sowie über den unabhängigen Kommentar des Wissenschaftlichen Beirats mit transdisziplinärem Gesundheitsfokus (Kapitel 8; [5, S. 458–606]).

Der Schwerpunktbeitrag in Kapitel 8 wurde aus Wissenschaftssicht zeitnah erweitert um transdisziplinäre Einblicke in die intensive Auseinandersetzung mit Corona-Erfahrungen in Politik und Praxis, den Fokus auf Einsamkeit sowie auf internationale und nationale Studien. Das BMAS unterstützte die entsprechende (Open Access) Buchpublikation *Gesundheit – Teilhabechancen – Diskriminierungsrisiken. Health in All Policies als Querschnittsaufgabe bei Beeinträchtigungen und Behinderung* [15].

### Staatenberichte und UN-Monitoring

Parallel zur nationalen Berichterstattung muss Deutschland regelmäßig an den Fachausschuss für die Rechte von Menschen mit Behinderungen der Vereinten Nationen berichten. Der Ausschuss prüft die Berichte und gibt Empfehlungen.

Im Jahr 2011 reichte Deutschland den ersten Staatenbericht zur Umsetzung der UN-BRK in Genf ein (Initialbericht). Nach vertiefenden Diskursen meldete sich im Mai 2015 der Ausschuss für die Rechte von Menschen mit Behinderungen (Fachausschuss) mit den „Concluding observations on the initial report of Germany“ [16].

Der Fachausschuss wies in seinen *Abschließenden Bemerkungen* (2015 und 2023) kritisch hin auf das *Fehlen eines menschenrechtlichen Behinderungsbegriffs*. Der grundlegende Mangel an klaren Zielvorgaben und Indikatoren zur Überwachung der Umsetzung des Übereinkommens wurde jeweils vermisst, ebenso wie *hinreichende Daten*, um Behinderung im Sinne der Konvention zu erfassen. Angemahnt wurden außerdem *mehr Transparenz* über Datensammlungen und Statistiken (Artikel 31) und mehr Ausgewogenheit im föderalen Skript der Konventionsentwicklung auf Länder- und kommunaler Ebene. Ein weiteres Anliegen war die stärkere Partizipation von Selbstvertretungsorganisationen. Schließlich wurde zu mehr Aufmerksamkeit für *Bewusstseinsbildung* und *zum* Einsatz für Zugänglichkeit und Teilhabe entlang der Artikel 5–30 UN-BRK eingeladen [2]. Insofern empfahl der Ausschuss mehr Partizipation und forderte Garantien für den Einklang aller künftigen Rechtsvorschriften und Konzepte mit dem Übereinkommen.

Die Aufforderung, ein menschenrechtliches, diskriminierungssensibles Modell von Behinderung als Maßstab zu nutzen, wurde im Bezug zum zweiten und dritten Staatenbericht der Bundesregierung vom September 2019 erheblich eindringlicher wiederholt und klar abgegrenzt von dem in vielen Rechtsbereichen auf Bundes- und Länderebene weiter üblichen medizinischen Modell. Diskriminierungsrisiken bei Intersektionen zu Behinderung wurden besonders beleuchtet und geeignetere Datenerhebungsmethoden angemahnt [17]. Es wird erwartet, dass die *Abschließenden Bemerkungen* aus dem Jahr 2023 derzeit auf Bundes‑, Landes- und kommunaler Ebene umgesetzt werden. Als Zwischenschritt wurde ein Maßnahmenbericht eingereicht (2023; CRPD/C/DEU/CO/2-3) und bis Ende Frühjahr 2031 werden im Rahmen weiterer Überprüfungsperioden der vierte, fünfte und sechste Prüfbericht fällig.

## Institutionelle Verantwortung und Akteure

In Tab. 3 sind die zentralen Akteure sowie deren Aufgaben und Bezugspunkte bei der Umsetzung der Teilhabeberichte skizziert.

**Tab. 3:** Übersicht der zentralen Akteure bei der Umsetzung der Teilhabeberichte

Akteur	Funktion und Aufgabe
*Bundesministerium für Arbeit und Soziales (BMAS)*	Federführung für die Umsetzung der Behindertenrechtskonvention der Vereinten Nationen (UN-BRK; National Focal Point); Herausgeber der Teilhabeberichte; Auftraggeber des Teilhabesurveys
*Monitoring-Stelle UN-BRK* (am Deutschen Institut für Menschenrechte)	Unabhängige Überwachung der Umsetzung der UN-BRK in Deutschland
*Wissenschaftlicher Beirat*	Multidisziplinäres Gremium; steuert unabhängig wissenschaftliche Analysen und Empfehlungen zu den Teilhabeberichten bei (insbesondere mit Bezug zu den Lebensbereichen und Schwerpunkten)
*Inklusionsbeirat*	Beratungsgremium aus der Zivilgesellschaft; vertritt Interessen von Menschen mit Behinderung
*UN-Fachausschuss für die Rechte von Menschen mit Behinderungen*	Internationales Gremium; prüft Staatenberichte und gibt Empfehlungen
*Länder und Kommunen*	Zuständig für Umsetzung in vielen Lebensbereichen (Bildung, Wohnen, Nahverkehr); entwickeln eigene Aktionspläne
*Rehabilitationsträger und Eingliederungshilfe*	Leistungserbringer; setzen Teilhabeleistungen konkret um und vertreten Interessen
*Selbstvertretungsorganisationen und Verbände*	Interessenvertretung; Partizipation an Planung und Evaluation

### BMAS als federführende Stelle.

Das BMAS ist die zentrale Koordinierungsstelle für die Umsetzung der UN-BRK auf Bundesebene. Es gibt die Teilhabeberichte heraus, beauftragt den Teilhabesurvey und koordiniert die Ressortabstimmung. Diese flankiert mit internen, übergreifenden Kommentierungen nach gesetzlichen, rechtlichen, politischen, gesundheits- und präventiven bundesdeutschen Anliegen die Berichte intern mit spezifischen Wissensbeständen.

Das BMAS arbeitete eng mit einem multidisziplinären Wissenschaftlichen Beirat zusammen, der die veröffentlichten Berichte fachlich begleitete und unabhängig Kommentare beisteuerte [3, S. 9]. Die zuständige Bundesministerin Ursula von der Leyen stellte diesen Beirat aus Wissenschaftlerinnen und Wissenschaftlern ohne und mit eigener Behinderungsexpertise der neuen Teilhabeberichterstattung zur Seite. Er steuerte jeweils in Berichtsteilen fachbezogen Daten, Analysen und Bewertungen bei, gab handlungsleitende Empfehlungen und wies auf relevante Datenlücken hin.

So sollten wissenschaftliche Qualität und politische Handhabbarkeit verbunden werden. Der Vierte Teilhabebericht liegt im Entwurf vor; eine unabhängige wissenschaftliche Kommentierung der Teilhabeberichterstattung ist für diesen Bericht nicht mehr vorgesehen.

### Monitoring.

Die Monitoring-Stelle am Deutschen Institut für Menschenrechte überwacht unabhängig, ob Deutschland die UN-BRK umsetzt. Sie veröffentlicht eigene Berichte, gibt Empfehlungen und nimmt Stellung zu gesetzlichen Vorhaben. Ihre Unabhängigkeit ist durch die Pariser Prinzipien der Vereinten Nationen gesichert.

### Föderale Fragmentierung.

Die Umsetzung der UN-BRK ist in Deutschland durch die föderale Struktur fragmentiert. Viele Teilhabebereiche – insbesondere Bildung – liegen in der Zuständigkeit der Länder. Die Entwicklung von Landesaktionsplänen und regionalen Maßnahmen verläuft bisher uneinheitlich, was der UN-Fachausschuss wiederholt kritisiert hat.

## Methodische Entwicklung und Datenprobleme

### Datenprobleme: Warum bisherige Statistiken nicht ausreichen

Die Teilhabeberichterstattung stößt auf grundlegende Datenprobleme:

1. *Amtliche Statistiken* erfassen vor allem Menschen mit anerkannter (Schwer‑)Behinderung – also jene, die einen Antrag gestellt und einen Bescheid erhalten haben. Menschen mit Beeinträchtigungen ohne amtliche Anerkennung bleiben weitgehend unsichtbar.
2. *Unterschiedliche Erhebungskonzepte* führen zu stark abweichenden Zahlen: Je nach Definition und Methodik (Erhebungsform und Zuschnitt) können Angaben zur Anzahl von Menschen mit Behinderung erheblich variieren. Analoges gilt für Daten zur Häufigkeit von Arten und Intersektionen von Beeinträchtigungen. Das trübt Erkenntnisgewinne.
3. *Bestehende Surveys* wie das Sozio-oekonomische Panel (SOEP) sind auf Privathaushalte zugeschnitten. Menschen in Einrichtungen der Eingliederungshilfe oder Pflege werden nicht erfasst. Das SOEP bietet zwar seit den 1980ern eine multidisziplinär aufgebaute strukturbezogene Datenbasis zum „Leben in Deutschland“, aber nach Teilhabechancen bei Behinderung zu fragen war nicht vorgesehen [3, S. 41 f.].
4. *Fehlende Verlaufsdaten:* Um Wirkungen von Maßnahmen zu beurteilen, sind Längsschnittdaten notwendig, die zeigen, wie sich Teilhabe über die Zeit entwickelt.
5. *Intersektionale Lücken:* Daten zu Mehrfachdiskriminierung (etwa Behinderung und Migration oder Behinderung und Geschlecht) sind kaum verfügbar.

Mit wachsender Präzision der Wissenslücken vertiefen sich nun die Gräben zwischen Näherungen zum UN-Antidiskriminierungsübereinkommen und dem Bedarf an erforderlichen Daten, weil bevölkerungsbezogene, regelmäßige Erhebungen (wie amtliche Statistiken, Panel-Studien bzw. Surveys) dazu fehlen [14].

Obwohl seit dem Jahr 1994 ein grundgesetzlich verankertes Benachteiligungsverbot bei Behinderung gilt (GG, Artikel 3, Absatz 3), blieb diese Datenkluft bestehen. Um Chancengleichheit und Chancengerechtigkeit als Grundrecht und über Wertentscheidungen ins Spiel zu bringen, ist aber informiertes Diversity Management gefragt, das mit nachvollziehbaren Unterscheidungen und differenzierten Leistungen Benachteiligungen wirksam abbaut. Deswegen muss sich auch datenbasiert neues Behinderungswissen formen.

### Der Lebenslagenansatz

Die Teilhabeberichte nutzen den *Lebenslagenansatz* als konzeptionellen Rahmen [3–5]. Dieser betrachtet Teilhabe nicht isoliert, sondern im Zusammenhang verschiedener Lebensbereiche: Wer in einem Bereich (etwa Bildung) benachteiligt ist, hat oft auch in anderen Bereichen (Arbeit, Einkommen, Gesundheit) schlechtere Chancen. Der Ansatz ermöglicht es, kumulative Benachteiligungen sichtbar zu machen und die Wechselwirkungen zwischen Lebensbereichen zu analysieren. Er ist anschlussfähig an die ICF, die ebenfalls Kontextfaktoren einbezieht.

Es geht entsprechend explizit auch um Entwicklungen der Teilhabe „am Leben in der Gesellschaft“, Merkposten für Diversitätsdimensionen wie „Gender Mainstreaming, Migration, Alter, Barrierefreiheit, Diskriminierung, Assistenzbedarf und Armut“ werden hervorgehoben und Bezüge zu Forschungsergebnissen „über Wirtschaftlichkeit und Wirksamkeit staatlicher Maßnahmen und der Leistungen der Rehabilitationsträger für die Zielgruppen des Berichts“ hergestellt [3].

### Der Teilhabesurvey

Um die Datenlücken bedarfsgerecht zu schließen, liefen zwischen 2011 und 2016 wissenschaftliche Vorstudien, um eine repräsentative Teilhabebefragung vorzubereiten [18]. Aspekte erprobter einschlägiger Repräsentativbefragungen wurden ebenso geprüft [19] wie mögliche Limitationen [20, S. 1113] und der Umgang mit besonderen Settings [21].

Die Bundesregierung wird über die umfassende „Repräsentativbefragung zur Teilhabe von Menschen mit Behinderungen“ (Teilhabesurvey) eine neue Datenbasis gewinnen. Das sollte ihre Fähigkeiten in Behinderungsfragen verbessern.

Die Differenzierung zwischen Beeinträchtigungen und Behinderung wurde bereits umgesetzt als Auftakt für künftig zuverlässigere Wissensbestände zur Lebenswirklichkeit von Menschen mit Behinderung. Im Survey werden zentrale Unterscheidungen getroffen zwischen

- Menschen mit Beeinträchtigungen,
- Menschen ohne Beeinträchtigungen,
- Menschen mit selbst eingeschätzter Behinderung.

Diese Substrukturierung erweitert die Erkenntnis und Analyse von Teilhabeunterschieden über die enge amtliche Definition hinaus.

Das beauftragte infas Institut für angewandte Sozialwissenschaft, Bonn, samt hinzugezogener weiterer wissenschaftlicher Partner, sollte

- die Lebenssituation von Menschen mit und ohne Beeinträchtigungen repräsentativ erfassen,
- Menschen in Einrichtungen (besondere Wohnformen, Pflegeeinrichtungen) einbeziehen, ebenso wie
- schwer erreichbare oder befragbare Personengruppen,
- Beeinträchtigungen und Behinderung im Einklang mit der UN-BRK und der ICF operationalisieren und
- quantitative und qualitative Methoden kombinieren.

#### Erste Welle des Teilhabesurveys.

Zwischen 2017 und 2020 ging der Teilhabesurvey an den Start. Er umfasste eine Bevölkerungsstichprobe (ca. 22.000 Personen ab 16 Jahren in Privathaushalten mit und ohne Beeinträchtigungen), eine Einrichtungsstichprobe (über 3300 Personen in Einrichtungen der Eingliederungshilfe in besonderen Wohnformen und in der Pflege in Alten- und Senioreneinrichtungen) sowie ergänzende Erhebungen zu schwer erreichbaren oder befragbaren Personengruppen.

Ein erster Abschlussbericht resümiert Datenlage und konzeptionelle Fragen mit einem Gesamtüberblick zu Befragungspotenzial und Methodik [22, 23]. Die mit großer Sorgfalt entwickelten Stichproben- und Befragungskonzepte verbinden quantitativ und qualitativ ausgerichtete Elemente [24, 25].

#### Zweite Welle des Teilhabesurveys.

In den Jahren 2023/2024 folgte eine erste Wiederholung der „Repräsentativbefragung zur Teilhabe von Menschen mit Behinderungen“ („Welle 2“; [25]). Nun wird vom Teilhabesurvey gesprochen, auch wenn es sich um keine für Panel typische Eins-zu-eins-Fortschreibung der „Welle 1“ handelt. Stattdessen wurden auf Wunsch des BMAS erste Befunde verstetigt, vertieft und validiert, aber auch neue Module ergänzt bzw. spezifisch erfahrungsbasiert verändert (z. B. Untertitel bei Bildung und lebenslangem Lernen; Beeinträchtigung und Gesundheit; persönliche Sicherheit; Partnerschaft und Familie). Daraus folgt ein Datenbestand im Mix aus Messung und Wiederholungsmessung. Zu Zuschnitt und Methodik (etwa der qualitativen Interviews oder zum modifizierten Panelansatz) und über zentrale Ergebnisse liegt ein erster Bericht vor [25]. Die Befragungsdaten der „Welle 2“ sollen über den Vierten Teilhabebericht zur Verfügung gestellt werden. Eine abschließende intensivierte Auswertung der qualitativen Daten zur „Welle 2“ steht noch aus.

Zu versorgungs- (Provider-centered) und nutzerorientierten (Consumer-centered) Leistungsformaten wird jeweils Wissen gewonnen, wie es die Leitlinien der UN-BRK nahelegen. Dabei bietet die international anschlussfähige, gemeinsam ausgestaltete (Shared Decision Making) ICF [9] Vorteile, weil mit ihr globale Diskurse zu Gesundheitsfragen im komplexen Zusammenwirken von Körperfunktionen und -strukturen, Aktivitäten, Partizipation sowie persönlichen und umweltbezogenen Kontextfaktoren konkret benannt werden können [10].

#### Grenzen des Teilhabesurveys.

Trotz des methodischen Fortschritts bleiben Einschränkungen:

- Die Welle 2 ist keine klassische Panelbefragung mit identischen Personen, sondern eine Wiederholungsbefragung mit modifizierten Modulen. Dies schränkt Längsschnittanalysen ein.
- Die abschließende Auswertung der ergänzenden qualitativen Interviews steht noch aus.
- Die Befragung kann nur erfassen, was Menschen berichten – nicht unbedingt, was sie objektiv erleben.
- Bestimmte Gruppen (etwa Menschen mit schweren kognitiven Beeinträchtigungen) bleiben auch mit angepassten Methoden schwer erreichbar.

#### Ergänzende Forschung während der Corona-Pandemie.

Parallel förderte das BMAS eine bundesweite Feldstudie zur Lage in besonderen Wohnformen während der Pandemie, zu Schutz- und Inklusionsanliegen unter der besonderen Risikolage. Dabei wurden alltagsnah Informationen zum Zusammenspiel der Sozialleistungsstrukturen nach SGB IX und der mit dem Gesundheitsschutz betrauten Behörden gewonnen. Beispielsweise zeigten sich ausgeprägte Diskrepanzen zwischen verschiedenen beteiligten Akteursgruppen bei der Wahrnehmung von Anordnungen, dem Verstehen von Vorgaben und deren Umsetzung [26, S. 94]. Dies legt nahe, bei Teilhabefragen die Partizipation unterschiedlicher Expertisen zu suchen.

## Inhaltliche Spannungen: Behinderungsverständnisse und das Inklusionsdilemma

### Drei Modelle von Behinderung

In der Diskussion um Behinderung konkurrieren verschiedene Verständnisse, die auch die Berichterstattung prägen. Unterschieden werden das medizinische, das soziale und das menschenrechtliche Modell von Behinderung (Tab. 4). Die UN-BRK folgt dem menschenrechtlichen Modell, das auf dem *sozialen Modell* aufbaut. Behinderung soll nicht als individuelle Abweichung, sondern als Ergebnis gesellschaftlicher Verhältnisse verstanden werden.

**Tab. 4:** Konkurrierende Modelle zum Verständnis von Behinderung

Modell	Kernaussage	Fokus
*Medizinisches Modell*	Behinderung gilt als ärztlich attestiertes individuelles Defizit, das nach Diagnose behandelt oder kompensiert werden muss	Person und ihre Einschränkung
*Soziales Modell*	Behinderung entsteht durch gesellschaftliche Barrieren, die benachteiligen und Teilhabe verhindern	Gesellschaft und ihre Strukturen
*Menschenrechtliches Modell*	Menschen mit Behinderung haben unveräußerliche Rechte auf Teilhabe und Selbstbestimmung	Rechte und Diskriminierungsschutz

In der deutschen Rechtspraxis dominiert vielfach das medizinische Modell – etwa bei der Feststellung von Behinderung durch ärztliche Gutachten oder bei der Orientierung an Diagnosen. Der UN-Fachausschuss hat dies wiederholt kritisiert. Die vorgeschlagenen Verbesserungen legen es nahe, international gebräuchliche Definitionen von Beeinträchtigungen und Behinderung als Maßeinheit zu nutzen, wie es bereits in den Teilhabeberichten angelegt ist, ergänzt um laufende menschenrechtliche, aber auch fachwissenschaftliche Diskurse zu Methodologien der Behinderungsforschung [27].

### Das Inklusionsdilemma: Differenz benennen, ohne zu diskriminieren

Inklusion zielt auf eine Gesellschaft, in der alle Menschen selbstverständlich dazugehören [28]. Zugleich erfordert Teilhabeberichterstattung, Unterschiede zu benennen: Ohne Daten über Menschen mit Beeinträchtigungen lässt sich nicht feststellen, ob oder wieweit sie gleichberechtigt teilhaben.

Dieses Spannungsverhältnis zeigt sich auf mehreren Ebenen:

1. *Kategorisierung und Stigmatisierung:* Die Zuordnung zu einer Gruppe „Menschen mit Behinderung“ kann Stigmatisierung verstärken, auch wenn sie dem Diskriminierungsschutz dient.
2. *Gleichbehandlung und Ungleichbehandlung:* Gleiches gleich zu behandeln kann ungerecht sein, wenn Ausgangsbedingungen verschieden sind. Inklusion erfordert oft, Ungleiches ungleich zu behandeln, um Benachteiligungen auszugleichen [28]. Hierzu fehlen allerdings Routinen für Individualisierung, wie die strittige Entwicklung rund um „Persönliche Budgets“ zeigt [29].
3. *Selbstidentifikation und Fremdzuschreibung:* Wer als behindert gilt, wird teils selbst entschieden (Selbsteinschätzung), teils von außen zugeschrieben (amtliche Feststellung, soziale Wahrnehmung). Diese Zugehörigkeit zu „den Behinderten“ bleibt im Sinn einer behördlichen Feststellung ebenso unscharf wie die, gemessen an vorgegebenen Leitkonzepten von Körperbeschaffenheit, Funktionalität oder Klassifikation. Die Zuordnungen überschneiden sich zudem mit der alltagsweltlichen Gewohnheit, Verhalten, Attraktivität oder Alters- und Generationenzugehörigkeit eher wie Eigenschaften wahrzunehmen [14, S. 10 f.].
4. *Heterogenität der Gruppe:* Menschen mit Behinderung bilden keine homogene Gruppe. Die Unterschiede nach Art der jeweiligen Beeinträchtigungen, Alter, Geschlecht, Herkunft und weiteren Dimensionen sind erheblich. Eine einheitliche Interessenvertretung als „kollektiver Körper“ „der Behinderten“ ist daher schwierig. Dass es außerdem keine einheitliche Definition von Inklusion gibt, erschwert die Kommunikation und Identifikation von Themenfeldern. Da Inklusion allerdings alle einbezieht, ist jede sichere Distanz ausgeschlossen.

Komplexe Spannungen bei dem Versuch einer Kategorisierung von Menschen mit Behinderung sind sachlogisch und locken zu Stereotypen über sie. Das erschwert zusätzlich ihr Selbstverständnis als „Wir“, als ein kollektiver Körper, der Allianzen gegen Diskriminierung schließen kann [28, S. 129].

### Normalisierung, Empowerment und Dekonstruktion

In der Inklusionsdiskussion lassen sich drei Grundhaltungen unterscheiden:

- *Normalisierung (N):* Integration in bestehende Strukturen durch Anpassung – das Ziel ist, so zu leben wie Menschen ohne Behinderung. Normalisierung meint vorrangig Integrationspfade, auf denen sich Verhalten und Verhältnisse bis zur Passung einfügen sollen.
- *Empowerment (E):* Stärkung der Rechte und Handlungsfähigkeit von Menschen mit Behinderung, auch durch Selbstorganisation und Interessenvertretung – Kampfkraft für eigene Rechte oder die anderer.
- *Dekonstruktion (D):* Infragestellung der Unterscheidung zwischen „normal“ und „behindert“; selbstbewusste Ablehnung einer Zuschreibung als „anders“ – Dekonstruktion von Normalismen, was Berichten über Differenzen herausfordert und an Grenzen bringt.

Diese Perspektiven ergänzen sich, stehen aber auch in Spannung zueinander. Insgesamt entpuppt sich das Verfassen von Berichten als spannende und spannungsreiche Kunst, bei der es gilt, mit Dissonanzen und Paradoxien umzugehen [32]. Allen Ambivalenzen zu folgen oder zu trotzen ist kaum möglich. Aber bestehende Barrieren lassen sich aufdecken. Das erlaubt, Zuschreibungen bei Behinderung kritisch zu betrachten, und hält davon ab, Normalisierung per se als ordnungsfördernd zu gewichten.

Berichte, die Inklusion fördern wollen, müssen mit diesen Ambivalenzen umgehen: Sie müssen Unterschiede benennen, ohne sie festzuschreiben, und Rechte stärken, ohne Gruppen zu essenzialisieren. Statt gegen Diskriminierung oft Einzelfälle (Cases) mit belegbarer Benachteiligung zu diskutieren, während Ursachen (Causes) im Hintergrund bleiben, können Teilhabeberichte dafür sensibilisieren (Awareness Raising), dass Inklusion das Erkämpfen von Rechten, die Beteiligung an einer sich verändernden Normalität oder die Ablehnung einer Zuordnung als „anders“ einschließt.

### Von Behinderungsvielfalt und sperriger Inklusion

Eingliederungshilfe soll Teilhaberechte bei Behinderung sicherstellen, ebenso wie eine inklusive Gesellschaft für alle. Gelingt dies, geraten Schutzzonen mit besonderer Unterstützung über Abgrenzung zu Recht auf den Prüfstand. Wenn Menschen mit Beeinträchtigungen und Behinderung angemessener sichtbar werden, können sie sich auch als Beteiligte gesellschaftlicher Veränderungen zeigen und als unterschiedlich – nach Dimensionen wie Alter, geschlechtliche Identität, Herkunft, körperliche und geistige Fähigkeiten, Religion und Weltanschauung, sexuelle Orientierung, soziale Herkunft und weiteren Vielfaltsaspekten. Berichte machen Unterschiede besser erkennbar und verständlich. Sie lassen allerdings auch Einblicke in Schutzräume zu und machen bestehende Strukturen hinterfragbar.

### Ringen um Klarheit mit Vielfaltsexpertise

Diskurse um Diversität und Inklusion bleiben voraussichtlich spannend und spannungsgeladen. Dies weckt Aufmerksamkeit für die Diversitätsdimension Behinderung in der Ungleichheitsforschung [33], fördert aber auch ein Inklusions-Diversitäts-Dilemma. Denn eine im Wettbewerb um Leistungen bislang vorteilhafte „Nische“ von Eingliederungshilfe und Rehabilitation gerät nun womöglich ins Rampenlicht von Spannungen zwischen Sozialem und Gesundheitssicherung.

Will man ernsthaft Teilhabeorientierung vermitteln, sollte dies jedoch nicht sorgen, denn Teilhabe entsteht durch Teilhabe. Kritische Vielfaltsfragen liegen als Preis des möglichst anpassungsfreien Eintritts in eine sich inklusionsorientiert verändernde Gesellschaft auf der Hand. Allerdings sind Kontrollverluste im etablierten Leistungssystem nicht auszuschließen, weil nun Themen der Zugehörigkeit zu Gruppen, die als behindert oder nicht behindert gelten, offen und zulässig sind [34, S. 18]. Differente Lebensweisen, Wissens- und Denkformen in ihrer eigenen Art (und Normalität) steigern demnach Komplexität, bei gleicher Anerkennung von Verschiedenheiten [35, S. 43]. Diese Büchse der Pandora lässt sich nach den menschenrechtlichen Maßgaben der UN-BRK aber ohnehin nicht mehr schließen.

Ein Lösungsweg ist Inklusivitätszuwachs jenseits der Fachspezifika, um breite Kompetenz im gesellschaftlichen Umgang mit Vielfalt aufzubauen. Dabei sind bei personenzentrierten Ansätzen Unschärfen ebenso üblich wie Risiken der Vielfalts-Vielfalt. Gesellschaftliche Einheit ohne Vereinheitlichung fordert heraus, kann aber über Diskurse, wie sie in demokratischen Gesellschaften an sich üblich sein sollten, moderiert werden. Dass dennoch Unmut über ausufernde Auseinandersetzungen um Anerkennung mit unkontrolliert steigenden Selbstbehauptungen erwacht [36], soll nicht verschwiegen werden. Denn letztlich gehen Inklusion und Individualisierung Hand in Hand [37, S. 294], was die klare Gestaltung von Chancengerechtigkeit und Diskriminierungsschutz kompliziert.

Individuen leben ihren Alltag ohnehin in unterschiedlichen Mitgliedschaften, parallel, kombiniert und als sich überkreuzende Mehrfachzugehörigkeiten, wenn sie nicht daran gehindert werden. Sie können sich zugleich als „Grannies“, Tennisspieler oder Verkehrsteilnehmerinnen verstehen, je nach situativer (Zu‑)Ordnung über wesentliche gesellschaftliche Strukturierungen bzw. Teilsysteme [38].

Soziologisch betrachtet ist dies wohlbekannt [39]. Wie fortschreitende Diversifizierungsprozesse Superdiversität hervorbringen, wird in komplexen Konfigurationen zwischen sozialer Kategorisierung und Organisation erforscht [40, 41].

## Bewertung: Leistungen, Grenzen und Perspektiven

Auch wenn die Teilhabeberichterstattung nicht mehr nur in den Kinderschuhen steckt, sondern sich bereits mit Kriterien ihrer Ausgestaltung befasst hat, so ist sie dennoch weit von qualitätsvoller Routine entfernt. Trotz der vorliegenden umfangreichen Berichte bleiben Datenlücken. Der Weg zu einer angemessenen Lebenslageneinschätzung bleibt verschlungen. Dennoch werden Fakten zur Lage der Teilhabe greifbarer und teilweise auch vergleichbarer. Zweifel werden differenzierter und Planungen besser argumentierbar.

An Grenzen stößt die Teilhabeberichterstattung, sobald man schnelle Wirksamkeit erwartet. Aber bereits der Auftrag, für Teilhabe zu sensibilisieren, ist ein Gewinn. Das Anliegen, Längsschnittdaten zu nutzen, ist in die Umsetzungsphase gelangt. Es erforderte ein komplexes Studiendesign, Kenntnisse dazu, wie man mit Vielfalt umgehen kann, und eine nachhaltige und auskömmliche Finanzierung. Dies alles befindet sich weiterhin im Baustellenmodus. Die Qualität der Datensätze und ihrer Nutzung ist ohnehin laufend zu sichern und zu bewerten, denn erst dann können sie nachhaltig zu Datengrundlagen werden.

### Umformtechniken – über Baustellen zum Umdenken

Umbauen braucht Zeit und eine kluge Suche nach bestmöglichen Lösungen. Dies gilt auch für die Aufgabe, neu über Behinderung, gesellschaftliche Teilhabe und Inklusionsentwicklung zu berichten. Man muss mit vielfachen Unschärfen umgehen und Gratwanderungen wagen, obwohl der Wunsch nach Klarheit eher zu riskanten Kategorisierungen lockt. Aber ungenaue Sachverhalte ermutigen auch zur kreativen Betrachtung.

Die im SGB IX enthaltenen Signale von Selbstbestimmung und Eigenverantwortung zu beachten rückt Teilhabefähigkeiten (Abilities) von Individuen (Personenbezug) ins Licht. Diese Signaletik in Leistungen zu transformieren, die zu nationalen und föderalen Filtern passen (Machbarkeit), bleibt allerdings herausfordernd.

Die Anliegen der internationalen und menschenrechtlich geprägten Datenvergleichbarkeit können einerseits Antrieb bieten, andererseits aber zu Schaukelkursen führen. Dies lässt sich über unabhängige, theoretisch fundierte und methodologisch ausgewogene wissenschaftliche Analysen nachvollziehen. Die Politik lenkt eher zweckgerichtet, pragmatisch und pflichtgemäß. Hier liegen Allianzen nahe, auch für Diskurse mit dem reich bestückten Feld der Stakeholder.

Eine 31-jährige Befragte mit Beeinträchtigungen fasst in der ersten Welle 1 der Teilhabebefragung ihr Anerkennungsanliegen und ihre Zweifel an Umformtechniken in eigene Worte [23, S. 110]:

„Ich denke kaum, dass da jemand so einzeln was dran ändern kann. Ich denke, dass einfach das ganze Denken der Menschen sich ändern müsste. Dass dieses Denken, dass man perfekt sein muss in der heutigen Welt, dass man nicht anders sein darf in der heutigen Welt, sich ändern müsste, und wenn man so den Verlauf ansieht und sich anguckt, dass in China jetzt schon perfekte Babys produziert werden sollen und dass in China entschieden werden darf, darf es ein Junge, darf es ein Mädchen sein, und solche Sachen halt eben, daran sieht man immer wieder, dass die Menschen eigentlich genau ins Gegenteil rennen. Anstatt dass sie Empathie für andere Menschen empfinden, laufen sie genau in das Gegensätzliche.“

### Resümee – Teilhabeberichte mit Wirkung im Wandel von Rehabilitation und Eingliederungshilfe

Teilhabeberichte, die es wagen, Unterschiedlichkeiten zu thematisieren, ohne Komplexität zu glätten, mögen unbequem sein. Womöglich hat dies der NAP nicht antizipiert. Aber sie können die Aufmerksamkeit auf erforderliche Ungleichheitsdiskurse lenken und auf allen gesellschaftlichen Ebenen auf mögliche Zuschreibungen oder nötige Zugehörigkeiten aufmerksam machen, die sich überlagern, verstärken oder relativieren. Ob dies nur irritiert oder Gleichstellungspolitiken dient, die unter Inklusionsdruck [36] Lebensumstände mit geringer Teilhabe (Poor Social Belonging) öffnen für einen besseren Zugang zu Ressourcen und Anerkennung, wird sich zeigen.

Es bestehen Chancen, dass Berichte Teilhabe verbessern, denn sie erhöhen Transparenz, ermöglichen informierte Debatten und können politischen Druck erzeugen. Wenn aber Berichte Teilhabelücken präziser benennen sowie Fortschritte und Rückschritte auf dem Weg zu wachsender Inklusivität besser erkennbar machen, müssen Taten folgen.

Insofern fordern Teilhabeberichte zu Recht Politik und Gesellschaft heraus. Aus wissenschaftlicher Perspektive ist *Umdenken* ohnehin *eine Daueraufgabe* die Erkenntnisgewinne hervorbringt. Aus politischer Sicht mag der *Umgang mit einer verkehrten Welt der Inklusivität* (statt der traditionellen sozialen Unterstützung) Mühe machen, zumal hier die eigene Berichterstattung den Spiegel vorhält.

Denn auch wenn gesellschaftlicher Zusammenhalt in Verschiedenheit letztlich nicht über Zahlenwerke geformt wird [27], können Daten doch zeigen, wenn Diskriminierung kein Einzelschicksal ist, sondern ein Ordnungsfaktor [37, 38, S. 319 f.].

Ob „Anders-Sein“ als problematisch gilt oder als genuiner Teil gesellschaftlicher Vielfalt, hängt mit dem intendierten oder bestehenden Gesellschaftsverständnis zusammen. Auch Teilhabeberichte spiegeln diese Verhältnisse. Den Umgang mit wachsender Komplexität zu meiden, ist keine Lösung.

## Data Availability

Alle dieser Arbeit zugrunde liegenden Daten sind in diesem Artikel enthalten.

## Footnotes

1 https://www.gemeinsam-einfach-machen.de/GEM/DE/AS/Home/as_node.html, zugegriffen: 01.03.2026.
